# Pathologic Changes in Wild Birds Infected with Highly Pathogenic Avian Influenza A(H5N8) Viruses, South Korea, 2014

**DOI:** 10.3201/eid2105.141967

**Published:** 2015-05

**Authors:** Hye-Ryoung Kim, Yong-Kuk Kwon, Il Jang, Youn-Jeong Lee, Hyun-Mi Kang, Eun-Kyoung Lee, Byung-Min Song, Hee-Soo Lee, Yi-Seok Joo, Kyung-Hyun Lee, Hyun-Kyoung Lee, Kang-Hyun Baek, You-Chan Bae

**Affiliations:** Animal and Plant Quarantine Agency, Anyang, South Korea

**Keywords:** highly pathogenic avian influenza, H5N8 subtype, migratory bird, pathological diagnosis, immunohistochemistry, South Korea, influenza, viruses, respiratory infections

## Abstract

In January 2014, an outbreak of infection with highly pathogenic avian influenza (HPAI) A(H5N8) virus began on a duck farm in South Korea and spread to other poultry farms nearby. During this outbreak, many sick or dead wild birds were found around habitats frequented by migratory birds. To determine the causes of death, we examined 771 wild bird carcasses and identified HPAI A(H5N8) virus in 167. Gross and histologic lesions were observed in pancreas, lung, brain, and kidney of Baikal teals, bean geese, and whooper swans but not mallard ducks. Such lesions are consistent with lethal HPAI A(H5N8) virus infection. However, some HPAI-positive birds had died of gunshot wounds, peritonitis, or agrochemical poisoning rather than virus infection. These findings suggest that susceptibility to HPAI A(H5N8) virus varies among species of migratory birds and that asymptomatic migratory birds could be carriers of this virus.

## Abstract 2

Susceptibility to infection varies by species, and asymptomatic birds could be carriers.

Highly pathogenic avian influenza (HPAI) A virus infection of gallinaceous birds (e.g., poultry) is associated with high morbidity and mortality rates (*1*). Wild waterfowl, including ducks, are natural reservoir hosts for influenza A viruses and play a role in virus ecology and propagation. However, since 2003, repeated outbreaks of HPAI virus subtype H5N1 infection have occurred in poultry flocks in several Southeast Asia countries, resulting in high mortality rates among domestic ducks and wild migratory birds (*2*). Several studies have raised concern about the spread of HPAI virus by migratory birds (*3*–*5*).

Within the past 10 years, 4 outbreaks of HPAI A(H5N1) have occurred in South Korea (during winter and spring); migratory birds were identified as putative vectors (*6*–*9*). In 2014, an outbreak of HPAI A(H5N8) in South Korea led to the culling of millions of domestic poultry. Hundreds of sick and dead wild birds were collected and tested, and the results confirmed HPAI A(H5N8) virus infection (*10*,*11*). Examining the pathologic changes caused by H5N8 virus infection in different wild bird species is essential for understanding their role in the spread of this highly infectious virus. We therefore examined many of the dead or sick wild birds collected during an outbreak of HPAI A(H5N8) virus during 2014 and report the gross and histologic findings and the patterns of virus antigen expression. We examined 8 Baikal teals, 3 bean geese, 1 whooper swan, and 2 mallard ducks naturally infected with HPAI A(H5N8) virus.

## Materials and Methods

### Samples 

**Figure 1:**
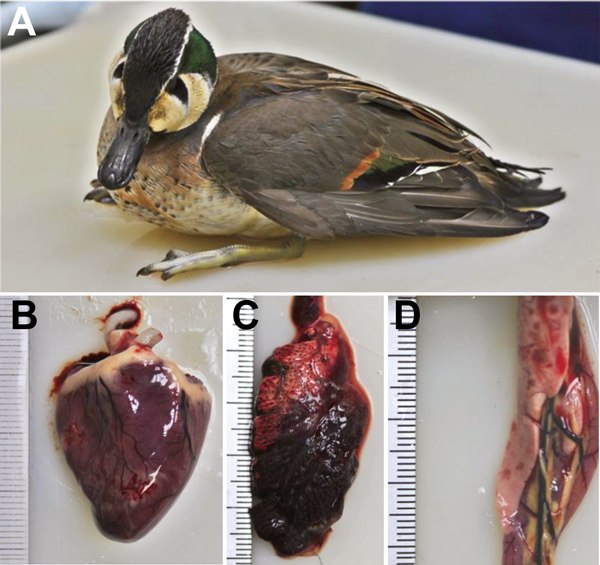
Baikal teal captured at Donglim Reservoir, showing A) neurologic signs of torticollis, ataxia, and limb paresis; B) hemorrhage and necrosis in heart muscle; C) edema and congestion of lung; and D) necrosis of pancreas.

During January–June 2014, a total of 771 wild bird carcassess were submitted to the Animal and Plant Quarantine Agency in Anyang, South Korea (Table 1). On January 17, many dead or sick wild birds were found around Donglim Reservoir in southwestern Korea. Three sick Baikal teals showing neurologic symptoms, including torticollis, ataxia, and limb paresis, were captured and euthanized (Figure 1, panel A). Over a 5-day period, the bodies of 119 Baikal teals, 9 bean geese, and 1 coot were collected from near the Donglim Reservior for necropsy. On January 22 and January 27, a total of 5 dead Baikal teals were found near the Geumgang River in midwestern South Korea (Table 2). Another 634 dead birds were found in other parts of the country. Necropsies were performed on all dead birds; trachea, kidney, cecal tonsil, pancreas, liver, intestine, heart, and lung were collected for virus isolation. Parenchymal tissues were collected for histopathologic analysis from 8 Baikal teals, 2 bean geese, and 1 whooper swan showing gross lesions and 3 bean geese and 2 mallard ducks not showing gross lesions. Collected tissues were fixed for 24 hours in 10% buffered neutral formaldehyde and processed for paraffin embedding. Bacterial culture was performed by using standard methods. The stomach contents were subjected to toxicology testing, as described previously (*12*).

**Table 1:** Results of necropsy of 771 bird carcasses collected January–June 2014, South Korea

Cause of death	Diagnosis	No. birds*
Pathogen (18.5%)	Highly pathogenic avian influenza A virus infection	167 (29)
	Bacterial infection	29
	Parasite infection	9 (1)
	Fungal infection	3
Nonpathogen (81.5%)	Agrochemical poisoning	222
	Gunshot	12
	Trauma	103
	Miscellaneous	32
	Putrefaction	52
	Unknown	142

*Parentheses indicate number of birds in which a pathogenic organism was detected in combination with agrochemical poisoning.

**Table 2:** Wild birds infected with highly pathogenic avian influenza A(H5N8) virus, South Korea, 2014*

Family/species	No. birds, n = 167	Region	Date	Gross lesions	Infected organs	Other cause of death
Anatidae						
Baikal teal (*Anas formosa*)	122	Donglim Reservoir	Jan 17–22	Y	T, C, K, L, Lu, P	None
	1	Jeonbuk	Jan 21	Y	P	None
	5	Geumgang River	Jan 22, 27	Y	T, C, K, Lu, P	None
	20	Chungnam	Jan 23	Y	T, K, P, H	Monocrotophos poisoning
Bean goose (*Anser fabalis*)	9	Donglim Reservoir	Jan 19–21	N	T (K)	Monocrotophos poisoning (*3*)
	1	Incheon	Feb 1	Y	UK	None
	1	Gyeonggi	Mar 9	Y	T, C, P	None
Mallard (*Anas platyrhychos*)	1	Jeonnam	Jan 27	N	UK	Peritonitis
	1	Jeonnam	Jan 29	N	UK	Gunshot, parasite infection
White-fronted goose (*Anser albifrons*)	1	Gyeonggi	Jan 28	N	UK	None
Whooper swan (*Cygnus cygnus*)	1	Jeonbuk	Feb 6	Y	UK	Renal failure
Other (not Anatidae)						
Coot (*Fulica atra*)	1	Donglim Reservoir	Jan 22	None	I, K	Postmortem change
Little grebe (*Podicdeps ruficollis*)	2	Gyeonggi	Feb 27	None	T, C, K	Postmortem change
Great egret (*Egretta alba alba*)	1	Jeonbuk	Mar 8	N	UK	Peritonitis

*C, cecal tonsil; H, heart; I, intestine, K, kidney; L, liver; Lu, lung; N, no; T, trachea P, pancreas; UK, unknown (pooled trachea, cecal tonsil, and kidney); Y, yes.

### Virus Isolation and Identification

Tissue samples from wild birds were inoculated into specific pathogen free embryonated chicken eggs (9–11 days of gestation), and influenza viruses were identified by using a hemagglutination assay and reverse transcription PCR. Virus identification was confirmed by sequence analysis, as described previously (*10*). In addition, molecular pathotyping was performed by nucleotide sequence analysis of the hemagglutinin cleavage site within the H5 subtype.

### Histopathology and Immunohistochemistry

Paraffin-embedded sections were cut (5 μm), dewaxed, and stained with hematoxylin and eosin. Duplicate sections were immunohistochemically analyzed to determine the distribution of influenza virus antigens in individual tissues. Briefly, sections were stained with a mouse monoclonal antibody against influenza A virus nucleoprotein (MCA-400; AbD Serotec, Duesseldorf, Germany), followed by a biotinylated goat anti-mouse IgG secondary antibody. Bound antibodies were detected with an avidin-biotin detection system (Ventana Medical Systems, Tucson, AZ, USA). The RedMap kit (Ventana Medical Systems) served as the substrate chromogen.

## Results

### Wild Bird Carcasses

Of a total of 771 wild birds, HPAI A(H5N8) viruses were isolated from 167. For the other 604 birds, test results for other avian influenza viruses were negative (Table 1). Bacterial (*Escherichia coli*, *Staphylococcus aureus*, and *Salmonella* Typhimurium), parasitic (nematodes, cestodes), and fungal infections were diagnosed for 29, 9, and 3 birds, respectively. We found that 73% of birds died of noninfectious causes. Agrochemicals, including monocrotophos, phosphamidon, carbofuran, diazinon, carbosulfan, endosulfan, parathion, dichlorvos, and methomyl, were found in the stomach contents of 222 birds; gunshot wounds, trauma (road kill or fracture), or miscellaneous (cachexia, dehydration, or suffocation) were the cause of death for 12, 103, and 32 birds, respectively. For 194 wild birds, the cause of death could not be determined because of postmortem autolysis, putrefaction, or both. 

### Observation of Gross Lesions and Isolation of HPAI Virus 

During January–March 2014, a total of 167 wild birds of 8 species were infected with HPAI A(H5N8) virus. All 148 infected Baikal teals showed evidence of multifocal necrosis in the pancreas and liver, pulmonary congestion and edema, subepicardial hemorrhage, and myocarditis (Figure 1, panels B–D), and H5N8 virus was isolated from the trachea, cecal tonsil, kidney, liver, lung, pancreas, and heart. Monocrotophos poisoning was also diagnosed for 20 Baikal teals collected in Chungnam Province. Although no lesions were visible in the organs of 9 bean geese found near Donglim Reservoir, H5N8 virus was identified in the trachea and kidney of all 9. These birds also contained high concentrations of monocrotophos. Necropsy of 2 bean geese and 1 whooper swan found during February–March revealed distinct lesions in the pancreas and kidney; H5N8 virus was isolated from the trachea, cecal tonsil, and pancreas. However, no gross lesions associated with HPAI virus infection were found in the organs from a mallard and a white-fronted goose; for these birds, the cause of death seemed to have been peritonitis and gunshot wounds. HPAI A(H5N8) virus infection was found in 1 coot, 2 little grebes, and 1 great egret; however, because of postmortem changes, no gross lesions associated with HPAI virus infection were identified (Table 2). All H5N8 virus isolates showed an HPAI virus motif (LREK[R]RRKR/GLF) at cleavage sites of hemagglutinin.

### Histopathologic and Immunohistochemical Findings

#### Baikal Teals

**Figure 2:**
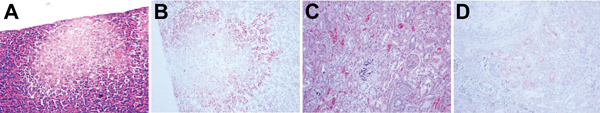
Histopathologic and immunohistochemical (IHC) testing results for Baikal teal. A) Focal necrosis in pancreas (hematoxylin and eosin [H&E] stain). B) Avian influenza virus antigen in necrotic pancreatic acini ([IHC stain). C) Gout and renal tubular necrosis (H&E stain). D) Avian influenza virus antigen in renal tubule cells (IHC stain). Original magnifications ×100.

Histologic examination revealed lesions in the pancreas, kidney, brain, and lung of all 8 birds examined. The pancreas showed moderate to severe, multifocal to confluent acinar necrosis, and virus antigen was detected in necrotic cells (Figure 2, panels A, B). Glomerular capillaries showed evidence of diffuse thrombosis and mild necrosis of tubules along with crystalline urate; virus antigen was detected in the tubular epithelium and glomerular capillary endothelium (Figure 2, panels C, D). Mild lymphocytic perivascular cuffing and loss of Purkinje cells were observed in the cerebrum and cerebellum, and virus antigen was detected in ependymal cells and epithelium of the choroid plexus and in cerebellar Purkinje cells. The lungs showed evidence of marked congestion, edema, and hemorrhage, and thrombosis was found in the alveolar capillaries. Influenza virus antigen was observed in a few capillary endothelial cells and macrophages in the alveolar lumen. Mild multifocal necrosis of hepatocytes and a lymphocytic infiltrate were also observed, and massive amounts of virus antigen were distributed within the sinusoidal endothelium and in necrotic hepatocytes within the liver. No lesions were visible in the trachea, intestine, muscle, spleen, or heart, and no antigen-positive cells were found (Table 3).

**Table 3:** Histopathologic lesions and immunohistochemical results for avian influenza virus antigen in 11 wild birds infected with highly pathogenic avian influenza virus

Organ	Positive result by histopathology/immunohistochemistry*			
	Baikal teal, 8/8	Bean goose, 2/2	Whooper swan, 1/1	Mallard, 0/0
Trachea	–/–	NT	NT	NT
Lung	+/+	+/++	NT	NT
Heart	–/±	+/++	–/–	–/–
Brain	+/++	+/++	+/++	NT
Kidney	+/++	+/++	++/++	NT
Skeletal muscle	–/–	NT	NT	NT
Intestine	–/–	–/–	–/–	–/–
Pancreas	+++/+++	++/+++	++/++	–/–
Liver	++/++	NT	±/+	NT
Spleen	–/–	–/–	+/–	NT
*Histopathologic results: –, no lesions; +, mild lesions; ++, moderate lesions, +++, severe lesions. Immunochemistry results: –, no antigen; ±, faint antigen; +, mild antigen; ++,moderate antigen; +++ severe antigen; NT, not tested.				

#### Bean Geese

**Figure 3:**
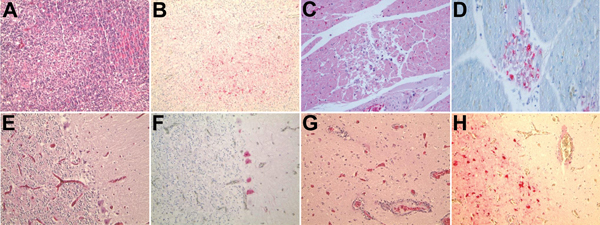
Histopathologic and immunohistochemical (IHC) testing results for bean goose. A) Diffuse necrotizing pancreatitis (hematoxylin and eosin [H&E] stain). B) Avian influenza virus antigen in necrotic pancreatic cells (IHC stain). C) Segmental necrosis of myofibers with mildly swollen nuclei focal necrosis (H&E stain). D) Avian influenza virus antigen in necrotic myofiber of the heart (IHC stain). E) Paucity of Purkinje cells, cerebellum (H&E stain). F) Avian influenza virus antigen in Purkinje cells (IHC stain). G) Neuronal necrosis and perivascular cuffing, cerebrum (H&E stain). H) Avian influenza virus antigen in neuron and glial cells (IHC stain). Original magnifications ×100.

In 2 of 3 bean geese examined, major histopathologic lesions were found in the same organs as in the Baikal teals. Moderate multifocal pancreatic necrosis was also observed. Myocardial myofibers showed evidence of segmental necrosis, and mildly swollen nuclei, focal necrosis, and virus antigen were detected in the heart (Figure 3, panels C, D). Also observed were randomly distributed foci of neuronal necrosis and mild to moderate lymphocytic perivascular cuffing in the cerebrum and a paucity of cerebellar Purkinje cells and focal necrosis in the cerebellum. Staining was positive for virus antigen in neurons, glial cells, dendritic cells, granule cells, and Purkinje cells (Figure 3, panels E–H). Moreover, renal tubular necrosis and crystalline urinary casts were observed in the kidney, and virus antigen was detected in the tubular epithelium. No lesions were evident in intestine, skeletal muscle, or spleen (Table 3).

#### Whooper Swan

In the 1 bird examined, lesions were found mainly in the pancreas, kidney, and brain. The distribution of the lesions and the antigenic staining patterns were similar to those observed for bean geese.

#### Mallard Duck

Of the 2 mallard ducks examined, a heterotopic parasite was observed in the pancreas of 1 and fibrinous peritonitis affecting the pancreas and intestine was observed in the other. No virus antigen was detected in the intestine or pancreas of either bird (Tables 2,3).

## Discussion

The 2014 outbreak of HPAI A(H5N8) in South Korea was unexpected because the H5N8 subtype is uncommon in this area. A genetic characterization study suggests that this H5N8 virus (clade 2.3.4.6) was introduced into South Korea by migratory birds and spread from there to poultry farms (*10*). 

Infection with H5N8 virus was found in all 148 Baikal teals, 2 bean geese, and 1 whooper swan. Necrotic lesions and avian influenza virus antigen staining were observed in multiple visceral organs, suggesting that the H5N8 virus causes a systemic infection. It also seems that the neurotropism of the H5N8 virus was the key factor contributing to death in these migratory birds of 3 species. The results of this study are consistent with those of other studies of HPAI pathogenicity in experimentally infected waterfowl (*3*–*5*,*13*). The gregarious behavior and migratory patterns of Baikal teals may underlie the mass mortality event that occurred at Donglim Reservoir.

Although a few Baikal teals were sick but not dead, the infection was clinically severe, and gross and histopathologic lesions were found. In addition, in 9 bean geese (all found in the same location at the same time), no evidence of lesions was found; however, the H5N8 virus was identified in the trachea and kidneys. Thus, the H5N8 virus did not cause sudden death in these waterfowl, despite their infection with the virus. This finding suggests that the infection is not peracute during the early stages.

By contrast, although mallard ducks and white-fronted geese were asymptomatically infected with H5N8 HPAI, these birds died of other causes, including gunshot wounds or peritonitis. Experimental infection studies show that some wild ducks, geese, and swans shed H5N1 virus despite being asymptomatic (*5*,*14*–*16*). Also, HPAI subtype H5 viruses have been isolated from healthy wild waterfowl, providing evidence of nonlethal infection (*17*,*18*). Thus, these species of migratory bird may be long-distance vectors for the H5N8 virus.

The histopathologic findings and the localization of H5N8 virus antigen associated with renal failure and gout in Baikal teals, bean geese, and whooper swans were unusual. Experimental infection studies have shown that although HPAI (H5N1) infects the tubular epithelium in the kidneys of various waterfowl, no evidence of gross or histopathologic lesions has been found in the kidneys (*4*,*5*). A few studies report that low pathogenicity H9 and H10 influenza viruses are nephrotropic in chickens (*19*,*20*) and that HPAI subtype H5 causes acute renal lesions in mammals and primates (including humans) (*21*–*23*). The results of our study suggest that the HPAI A(H5N8) virus affects waterfowl differently than do other HPAI viruses; therefore, further studies are needed to fully understand the pathology of H5N8 in waterfowl.

In summary, we report the pathogenicity of HPAI A(H5N8) virus (clade 2.3.4.6) in various species of waterfowl in South Korea. Baikal teals, bean geese, and whooper swans are susceptible to this virus, which causes high mortality rates; however, infection in mallard ducks is asymptomatic. Although many questions regarding HPAI A(H5N8) virus pathogenesis remain, the results reported herein suggest that susceptibility to HPAI A(H5N8) virus differs among different species of migratory birds. Thus, these birds may be susceptible to or carriers of this infectious virus.
